# Report on Vibrio Species Contamination in Shrimp From the Coast of Pangandaran, West Java, Indonesia

**DOI:** 10.1111/1758-2229.70210

**Published:** 2025-10-09

**Authors:** Titin Herawati, Indriyani Rahayu, Aisyah Aisyah, Mochamad Untung Kurnia Agung, Buntora Pasaribu, Atikah Nurhayati, Adiana Ghazali, Roffi Grandiosa, Thallita Nasywa Faddilah, Rendika Kamiswara

**Affiliations:** ^1^ Master of Marine Conservation Universitas Padjadjaran Sumedang Indonesia; ^2^ Department of Marine Science Universitas Padjadjaran Sumedang Indonesia; ^3^ Department of Fisheries Universitas Padjadjaran Sumedang Indonesia; ^4^ Faculty of Science & Marine Environment Universiti Malaysia Terengganu Kuala Nerus Malaysia

**Keywords:** identification, monitoring, Pangandaran regency, pathogenesis, vibrio bacteria

## Abstract

Bacterial infections in aquatic organisms pose a significant threat to shrimp aquaculture, often leading to production losses. In Pangandaran Regency, early shrimp harvesting is frequently practiced as a response to outbreaks. Previous studies have documented Vibrio and non‐Vibrio bacteria in pond water and sediments at five stations, but infections in shrimp tissues remain less explored. This study aimed to identify pathogenic Vibrio species in vannamei shrimp ( *Litopenaeus vannamei* ) and wild black tiger shrimp ( *Penaeus monodon* ), examine toxin genes, and quantify bacterial abundance. Samples were collected from five stations and analyzed using culture media, Gram staining and Polymerase Chain Reaction (PCR). *Vibrio parahaemolyticus* and *Vibrio alginolyticus* were detected at Stations 1 and 5, whereas only *V. alginolyticus* appeared at Stations 2, 3 and 4. Station 1 showed the highest Vibrio abundance (1.3767 × 10^6^ CFU/g), while Station 3 recorded the lowest (0.009 × 10^6^ CFU/g), with significant differences (*p* < 0.05). Non‐Vibrio bacteria dominated most stations, except at Station 1 where Vibrio species were predominant. Toxin gene analysis revealed toxR in some isolates, while tdh, trh, pirA and pirB were absent. These findings emphasize the importance of bacterial and genetic monitoring to improve disease surveillance and support sustainable shrimp aquaculture.

## Graphical abstract

Shrimp samples from Pangandaran, Indonesia, revealed contamination by *Vibrio parahaemolyticus* and *Vibrio alginolyticus* . Toxin gene screening showed the presence of *toxR* but the absence of highly pathogenic markers (*tdh, trh, pirA, pirB*). These findings highlight infection risks and the need for improved biosecurity in shrimp aquaculture.

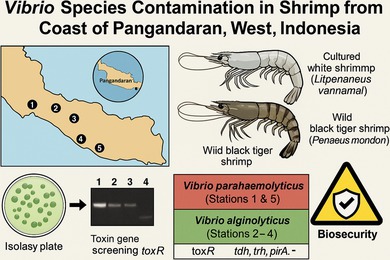

## 1 Introduction

Aquaculture is considered an essential industry providing global food security and nutrition. It is projected to contribute more than 60% of global aquatic animal production by 2030 (Kumar et al. 2021; Mok et al. 2021). Among several aquaculture industries, shrimp farming is in the spotlight sector due to the high market value and relatively short production cycles (Asche et al. 2021). Shrimp farming began expanding in Southeast Asia's coastal regions in the 1980s, exceeding wild‐caught shrimp in market share by 2007 (Jayasinghe et al. 2019). In Southeast Asia, shrimp have significantly contributed to the economies of Indonesia, the Philippines, Vietnam and Thailand, as well as Ecuador, Mexico and Brazil in the Americas (Manan and Ikhwanuddin 2018; Lightner 2011). The industry is still expanding on a large scale, with the majority of production occurring in East and Southeast Asia and Latin America, and most consumption in developed markets such as the United States, the European Union and Japan (Kumar et al. 2021).

Shrimp is decapod crustaceans belonging to the superfamily Penaeoidea, which is an important resource for both commercial fisheries and aquaculture worldwide, accounting for more than 30% of global crustacean consumption (Rosenberry 2001; Farfante and Kensley 1997). In the 1980s, aquaculture advanced through the intensification of the number of ponds and stocking densities of production units, complemented by advances in biotechnology that improved efficiency and quality across all production stages (Flegel 2019; Flegel et al. 2008). The development of the shrimp industry heavily relies on critical health management protocols (Khushi et al. 2022). Consequently, aquaculture diseases have significantly affected this sector by causing a decrease in the production of shrimp.

The *Vibrio* genus is a group of bacteria frequently associated with high mortality rates and significant economic losses in the aquaculture industry. To date, nearly 20 *Vibrio* species have been identified as pathogens responsible for causing diseases in aquatic animals (Joseph et al. 2015; Xu et al. 2017). Disease outbreaks in shrimp ponds often result from the complex interaction of multiple factors, including poor environmental conditions, high stocking densities combined with inadequate management, stress, suppression of the host immune system and infectious agents such as bacteria, viruses, fungi and parasites (Kennedy et al. 2016). One of the most impactful diseases affecting shrimp farming is bacterial infection, particularly vibriosis, which has been identified as a major threat to the success and economic sustainability of the industry (Hossain et al. 2017).

Vibriosis is caused by various *Vibrio* species, including *Vibrio harveyi* , *Vibrio alginolyticus* , *Vibrio parahaemolyticus* , *Vibrio vulnificus* and *Vibrio cholerae* , which can infect shrimp and lead to clinical symptoms such as stunted growth, low survival rates, reduced disease resistance, lethargy, tail cramping and erratic swimming behaviour, particularly under low salinity conditions (Ringø et al. 2020; Li et al. 2018). These infections often result in mass mortality, ultimately leading to production failure and significant economic losses in many Asian countries (Li et al. 2018).

Additionally, *Vibrio* spp. has been identified as a dominant microbe in shrimp hatcheries, affecting the reproductive cycle and causing mortality in both larvae and adult shrimp (Ahmmed et al. 2020; Khan and Mahmud 2020; Sumon et al. 2018). Furthermore, shrimp are also susceptible to various other pathogens, including *white spot virus* and *yellow head virus*, which further compromise their health and pose additional challenges to the sustainability of shrimp aquaculture.

Pangandaran Regency, West Java, with a 91 km coastline, hosts numerous shrimp ponds that contribute to local aquaculture and the regional economy. However, bacterial infections in these farming systems remain poorly studied, despite frequent premature harvesting due to suspected diseases. *Vibrio* spp. are of particular concern because they carry toxin genes such as *tdh*, *trh*, *pirA*, *pirB* and *toxR*, which play key roles in virulence. Detecting these genes is essential to assess pathogenic potential and disease risk. This study aimed to identify bacterial species, abundance and toxin gene presence as a basis for health management and preventive strategies across five coastal stations in Pangandaran.

## 2 Materials and Methods

### 2.1 Study Location

**FIGURE 1:**
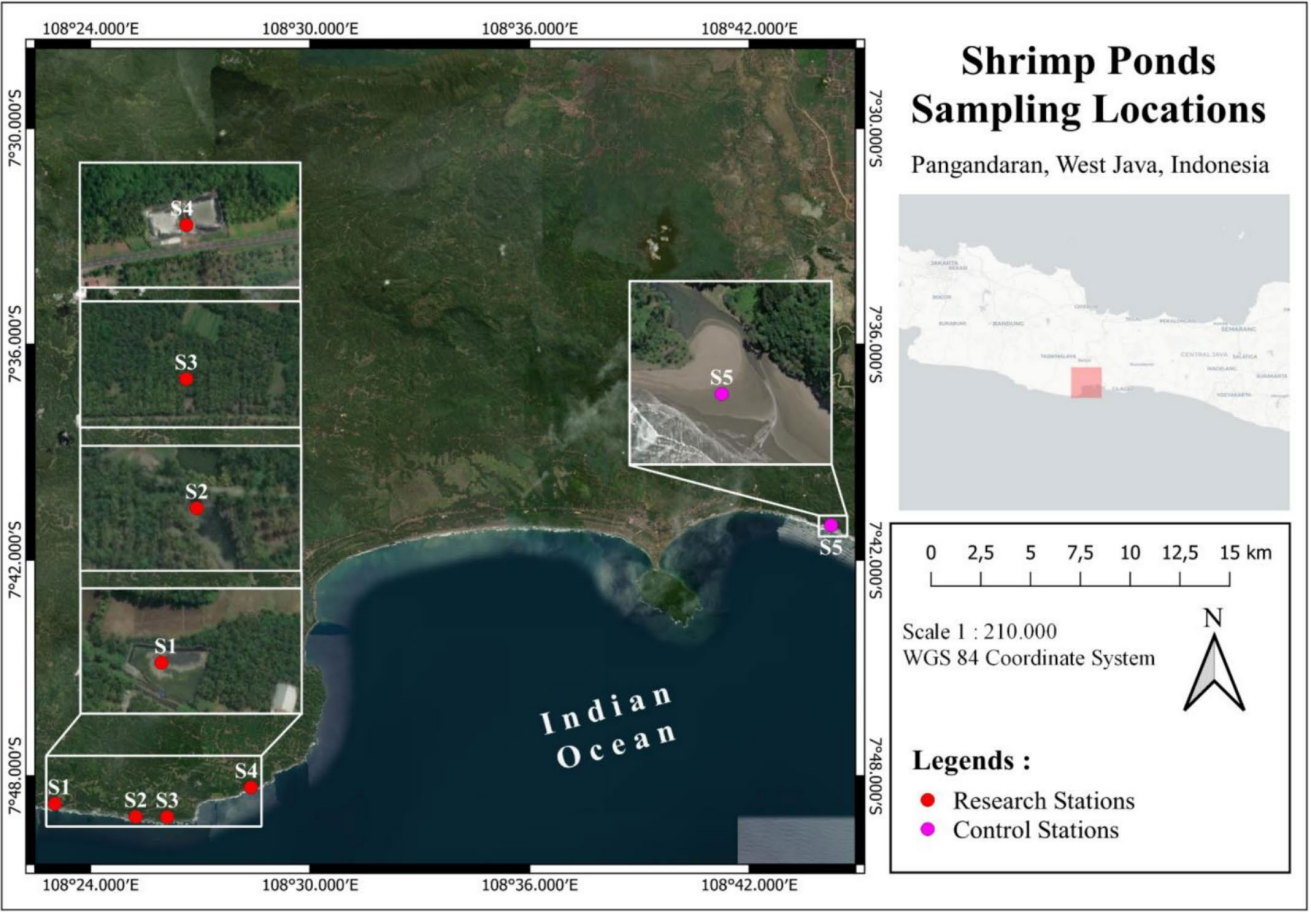
Study map station.

This study was conducted at five stations representing various shrimp farming systems along the southern coast of Pangandaran Regency, West Java. Station 1 is located in Kertamukti Village and is classified as a semi‐intensive shrimp pond, with coordinates at 7°48′46.23″S and 108°23′1.60″E. Stations 2 and 3 are situated in Legok Jawa Village, Cimerak District. Station 2 operates a semi‐intensive farming system (7°49′08.00″S, 108°25′14.20″E), while Station 3 is an intensive shrimp pond (7°49′8.50″S, 108°26′05.90″E). Station 4 is located in Madasari Village, also within Cimerak District, and employs an intensive farming system at coordinates 7°48′19.15″S and 108°28′22.98″E. Meanwhile, Station 5 is situated in the coastal waters of Karapyak Beach in Bagolo Village, Kalipucang District (7°41′1.851″S, 108°44′14.00″E) and serves as a control station, as no shrimp farming activities are present (Figure 1). The selection of these locations was intended to compare environmental conditions between semi‐intensive, intensive shrimp farming systems and the natural coastal environment unaffected by aquaculture.

The selection of study locations refers to the presence and absence of shrimp farming in pond activities and catches by fishermen around the coast. Stations 1, 2, 3 and 4 represent vannamei shrimp farming in ponds are located, while Station 5 is coastal waters where fishermen catch shrimp (Figure 1).

### 2.2 Sample Collection

Vannamei and black tiger shrimp samples were randomly collected at five stations during June and July 2024. A total of 5–10 shrimp from each station were sampled for general examination (Tran et al. 2013; Ananda Raja and Panigrahi 2012; NACA 2012). The samples were then processed by extracting portions of the carapace, swimming legs and tail using sterilised scissors. Subsequently, the samples were placed into sterile containers filled with 37.5 mL of sterile seawater mixed with 12.5 mL of sterile glycerol, securely sealed, properly labelled, and stored in a cool box containing ice cubes before being transported to the laboratory for microbiological analysis. All procedures were conducted in a well‐ventilated area while wearing sterile rubber gloves to prevent contamination (Bondad‐Reantaso et al. 2018, 2001).

### 2.3 Procedure Isolation, Culture, and Characteristics of Typical Colonies of *Vibrio* spp.

The bacterial isolation process was conducted using Thiosulfate Citrate Bile Salt Sucrose (TCBS) agar, which is specifically formulated for the isolation of Vibrio spp. due to its selective components such as thiosulfate, bile salts and sucrose. The typical formulation of TCBS per litre includes: yeast extract (5.0 g), enzymatic digest of casein (5.0 g), enzymatic digest of animal tissue (5.0 g), sodium citrate (10.0 g), sodium thiosulfate (10.0 g), bile salts (8.0 g), sucrose (20.0 g), sodium chloride (10.0 g), ferric citrate (1.0 g), bromothymol blue (0.04 g), thymol blue (0.04 g) and agar (14.0 g). The formula may be adjusted and/or supplemented as required to meet performance specifications (Microbiology International 2022). Prior to use, suspend 16 g of the medium in 200 mL of purified water, heat with frequent agitation, and boil for one minute until fully dissolved. The medium is then poured into sterile Petri dishes.

Samples were serially diluted from 10^−1^ to 10^−6^ and inoculated onto TCBS agar plates. The plates were incubated at 37°C for 24 h. After incubation, *Vibrio* colonies displayed specific morphological characteristics that facilitated identification. Colonies capable of fermenting sucrose appeared yellow with a diameter of approximately 2–3 mm and a bluish‐green centre. In contrast, non‐sucrose‐fermenting colonies appeared green or translucent. These typical colonies were counted using a colony counter to determine the concentration of *Vibrio* spp. in the samples.

Suspected *Vibrio* colonies were then subcultured by transferring them into sterile tubes containing Nutrient Broth (NB) and re‐incubated at 37°C for 24 h. Turbidity observed in the NB medium indicated successful bacterial growth. This culture was then used for further molecular analysis to obtain more accurate identification.

All isolation and culture procedures for *Vibrio* spp. followed international standards, namely ISO 21872‐1:^2017^ and ISO 21872‐2:^2007^.

### 2.4 Identification of Vibrio by Molecular Testing

The identification method was conducted based on Delidow et al. (1993). Samples were isolated using the Wizard Genomic DNA Purification Kit A1120 (Promega), and the Polymerase Chain Reaction (PCR) was carried out using specific primers. The primers used included 27F (AGAGTTTGATCCTGGCTCAG) and 1492R (TACGGYTACCTTGTTACGACTT) for the 16S rRNA gene, with a product size of 1460 bp (Lane 1991), as well as VF1 (AARCARGGNCGTAACCGTAA) and VR1 (HGGGTADCGRCGRCTCAT) for the gyrB gene, with a product size of 510 bp (Wang et al. 2024).

The tools used in the molecular testing were Thermal cycler PCR Sensoquest Genecraft Labs and Refrigerated Sigma 1‐14K centrifuge. Approximately 35 μL of reagents were used comprising 17.5 μL Green Taq Master Mix 2×, 11.9 μL of nuclease‐free water, 1.4 μL forward primer, 1.4 μL reverse primer and 2.8 μL DNA template. PCR settings for the P16s primers were 30 cycles with a pre‐denaturation step at 94°C for 90 s, denaturation at 95°C for 30 s, annealing at 48.5°C for 30 s and extension at 72°C. After completion, a final extension was performed at 72°C for 5 min. PCR conditions for gyrB were set with a pre‐denaturation at 94°C for 4 min, denaturation at 94°C for 1 min, annealing for 1 min, extension at 72°C for 1 min, for 35 cycles, a final extension at 72°C for 10 min, and storage at 4°C (Wang et al. 2024). Electrophoresis was performed using a Mupid EXu Submarine (Horizontal) Type Electrophoresis apparatus and visualized with a UV transilluminator UVITEC Cambridge.

PCR results were subjected to Single Pass DNA Sequencing at 1st Base DNA Sequencing. Furthermore, sequences obtained were analyzed using BioEdit and MEGA11: Molecular Evolutionary Genetics Analysis version 11 (Kumar et al. 2018). Subsequently, BLAST (https://blast.ncbi.nlm.nih.gov/Blast.cgi) and phylogenetic analysis were performed. The results were compared with existing genetic databases to identify homologous sequences and assess the evolutionary relationships among the samples. Potential new species were DNA sequences with similarity below the threshold of 98.65%. The arrangement of DNA sequence was conducted using MEGA, and a phylogenetic tree was constructed with the neighbour‐joining method for the isolates and related sequences. A total of 1000 bootstrap replicates were used in the construction of the phylogenetic tree.

### 2.5 Determination of Toxin Gene

Detection of toxin genes in *Vibrio* spp. isolates was performed using specific primers targeting the *tdh*, *trh*, *toxR* genes, as well as the *pirA* and *pirB* genes associated with Acute Hepatopancreatic Necrosis Disease (AHPND) (Table 1). PCR reactions were prepared in a total volume of 50 μL, consisting of 50 ng DNA template, 10 pmol of each primer, 25 μL of 2× PCR Master Mix (containing 2.4 mM dNTP and 0.3 units of Taq DNA polymerase; Promega, USA), and sterile distilled water.

**TABLE 1:** Primer sequences used for detection of toxin genes in *Vibrio* spp.

Gene	Primer name	Nucleotide sequence (5′ → 3′)	Product size (bp)	References
toxR	toxR‐F	GTCTTCTGACGCAATCGTTG	367	Luan et al. (2007) and Marlina et al. (2007)
	toxR‐R	ATACGAGTGGTTGCTGTCATG	367	Luan et al. (2007) and Marlina et al. (2007)
tdh	tdh‐F	GTAAAGGTCTCTGACTTTTGGAC	500	Luan et al. (2007) and Marlina et al. (2007)
	tdh‐R	TGGAATAGAACCTTCATCTTCACC	500	Luan et al. (2007) and Marlina et al. (2007)
trh	trh‐F	TTGGCTTCGATATTTTCAGTATCT	269	Luan et al. (2007) and Marlina et al. (2007)
	trh‐R	CATAACAAACATATGCCCATTTCC	269	Luan et al. (2007) and Marlina et al. (2007)
pirA	pirA‐F	ATGAGTAACAATATAAAACATGAAAC	284	Han et al. (2015)
	pirA‐R	GTGGTCGTTGAGATGTTGG	284	Han et al. (2015)
pirB	pirB‐F	TGACTATTCTCACGATTGGACTG	392	Han et al. (2015)
	pirB‐R	CACGACTAGCGCCATTGTTA	392	Han et al. (2015)

***Note:*** *tdh* = Thermostable direct hemolysin; *trh* = TDH‐related hemolysin; *toxR* = Toxin operon regulator (Luan et al. 2007; Marlina et al. 2007). *pirA* = Photorhabdus insect‐related binary toxin A, associated with AHPND; *pirB* = Photorhabdus insect‐related binary toxin B, associated with AHPND (Han et al. 2015).

Amplification was performed using an MJ Mini Thermal Cycler (Bio‐Rad, USA) under the following conditions: initial denaturation at 94°C for 3 min, followed by 30 cycles of denaturation at 94°C for 1 min, annealing for 1 min and extension at 72°C for 1 min. The annealing temperature was set according to the target gene, namely 50°C for *tdh*, *trh* and *toxR*, while 55°C–58°C was applied for *pirA* and *pirB*. The final extension was carried out at 72°C for 7 min.

PCR products were visualized by electrophoresis on 1% agarose gel using 1× TAE buffer, stained with ethidium bromide, and observed with a UV transilluminator system (DyNa Light).

### 2.6 Gram Staining and Colony Morphology Observations

Gram staining was performed to classify bacteria based on cell wall characteristics. This procedure was carried out according to Coico (2006) and started with the creation of a bacterial smear on a glass slide, which was fixed by heating. The sample was stained using crystal violet for 1 min, rinsed with water, and dripped into iodine solution for 1 min to form a complex. Decolourization was achieved with 95% ethanol, followed by counterstaining with safranin for 30 s. Gram‐positive bacteria appear purple, while Gram‐negative bacteria appear red or pink (Herawati, Rahayu, et al. 2025).

After incubation, colony morphology was documented based on physical characteristics such as shape (round, irregular), edge (smooth, wavy), elevation (flat, raised), colour and texture (smooth, rough, slimy). These observations provided additional insights into the bacterial genus or species. Table 2 is the reference for the colony colours of isolates grown on TCBS agar (Microbiology International 2022).

**TABLE 2:** Cultural response on TCBS Agar at 36°C–38°C after 24 h of incubation.

Microorganism	Response	Reaction
*Vibrio alginolyticus* ATCC 17,749	Growth	Yellow
Vibrio cholera ATCC 14,733	Growth	Yellow
*Vibrio furnissii* NCTC 11218	Growth	Yellow
*Vibrio parahaemolyticus* ATCC 10,885	Growth	Green

### 2.7 Enumeration of *Vibrio* Bacterial Abundance

Total *Vibrio* Count (TVC) and total plate count (TPC) are calculated using the following formula (Choudhary et al. 2025):

(1) $$\begin{array}{c} \text{Bacteria count}\;\left(\mathrm{CFU}\;{\mathrm{mL}}^{-1}\;\mathrm{dan}\;\mathrm{CFU}\;g^{-1}\right)\\ \;=\frac{\mathrm{no}.\;\text{of colonies}\times \text{dilution factor}}{\text{sample wight percentage}}. \end{array}$$

CFU = bacteria colony.

TVC refers to the total number of *Vibrio* colonies counted after incubation on TCBS media, while TPC counts the total number of colonies of all bacterial species present in the sample. By using these two metrics, the study can assess the number of *Vibrio* bacteria in shrimp farming environments or other tested samples (Choudhary et al. 2025).

### 2.8 Proportional Assessment of Vibrio and Non‐Vibrio Bacteria in Shrimp

A proportional analysis was conducted to compare *Vibrio* and non‐*Vibrio* bacteria in shrimp samples. *Vibrio* was isolated using selective TCBS media, while non‐*Vibrio* bacteria were isolated using non‐selective NA (Nutrient Agar) media. The isolation procedures for these two types of bacteria differ in the media used, with TCBS specifically designed for *Vibrio* and NA for non‐*Vibrio*. The *Vibrio* proportion was calculated by comparing colony counts on both media, providing a clearer picture of the bacterial composition in the samples. Formula 2 was used to measure the proportion of Bacteria Vibrio and non‐Vibrio (%) that could survive on TCBS and NA media (Boor et al. 2017; FSAI 2016; Gleeson et al. 2013):

(2) $$\text{Proportion bacteria}\;\left(\%\right)=\frac{\text{total bacteria}\;\mathrm{on}\;\text{TCBS media}\;}{\text{total bacteria}\;\mathrm{on}\;\mathrm{Na}\;\text{media}}\times 100$$

### 2.9 Statistical Analysis

This study employed ANOVA (Analysis of Variance) to compare the means of three or more groups and determine significant differences (Montgomery 2017). Prior to ANOVA, a homogeneity of variance test was conducted. If the data were not homogeneous, the Brown‐Forsythe or Welch ANOVA was used as a more robust alternative (Field and Wilcox 2017). Post hoc analysis using Tukey HSD was performed to identify specific group differences while effectively controlling Type I error (Juarros‐Basterretxea et al. 2024). All analyses were conducted using IBM SPSS Statistics version 27.

## 3 Result

**FIGURE 2:**
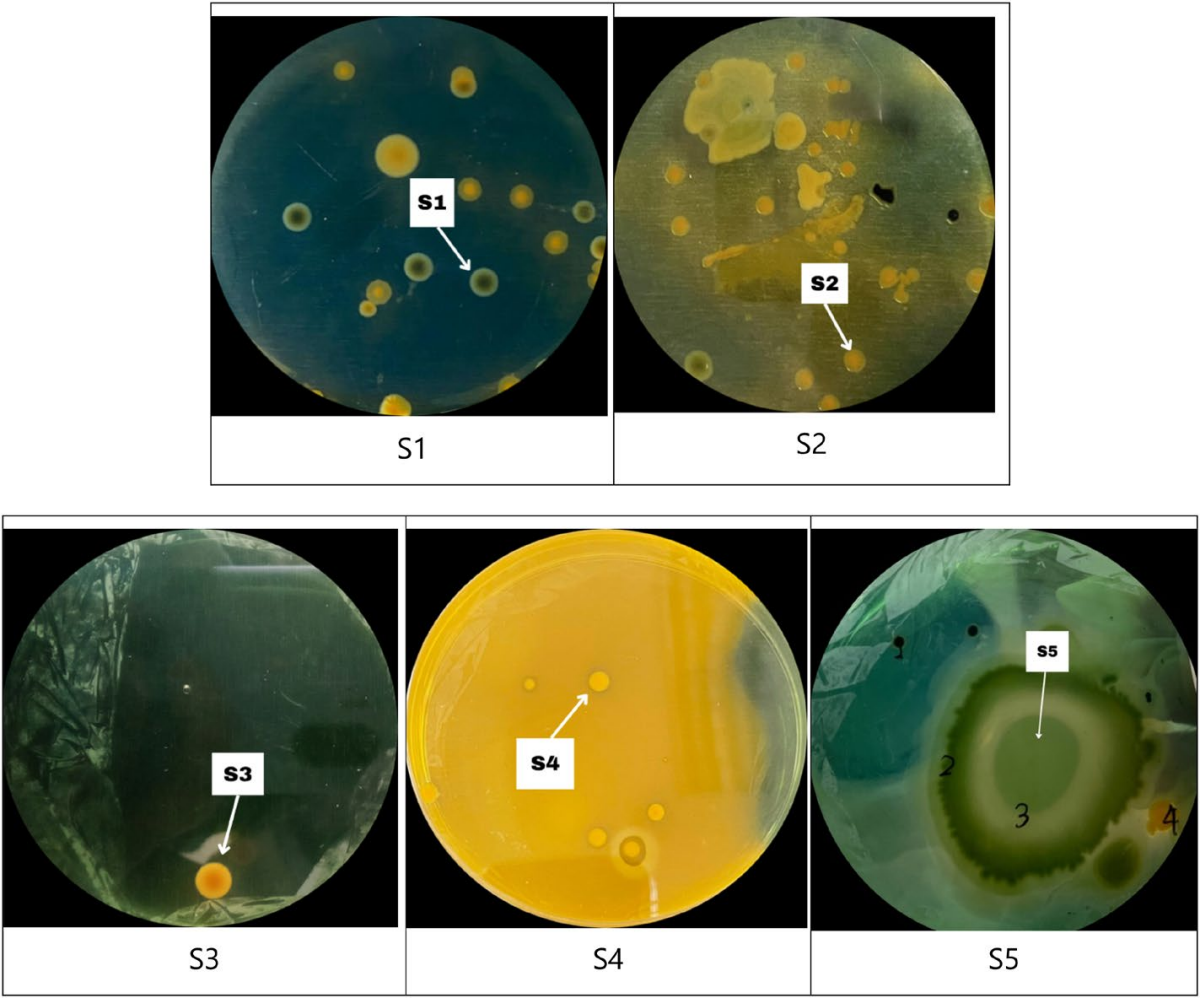
Appearance of bacterial colonies growing on TCBS media. Colonies marked with arrows were selected for further analysis based on colour variation and distinctive morphology.

The shrimp bacteria samples collected from five stations were grown in selective media, and Gram staining demonstrated the bacteria consisted of Gram‐negative, comma‐shaped bacteria, with colonies appearing green and yellow, as presented in Table 3 and Figure 2.

**TABLE 3:** Colony characteristics, cell morphology, and gram group of bacteria grown on TCBS medium.

Parameters	Station							
	1		2		3	4	5	
Colony								
Macroscopic appearance								
1. Colony colours								
Green	✓		✓				✓	
Yellow		✓		✓	✓	✓		✓
Microscopic appearance								
1. Cell Shaped								
Comma	✓	✓	✓	✓	✓	✓	✓	✓
2. Gram Group								
Negative	✓	✓	✓	✓	✓	✓	✓	✓

The colonies observed and analyzed in Table 2 were selected based on variations in colony colour (yellow and green) detected across all sampling stations, rather than from each station individually. Each selected colony represented specific morphological characteristics, both macroscopically (colony colour) and microscopically (cell shape and Gram staining), as observed on the growth media. These representative colonies, which are marked in Figure 2, were then analyzed using PCR and subjected to sequencing to determine the complete or partial DNA sequences with high accuracy, thereby enabling precise identification of the bacterial species.

**FIGURE 3:**
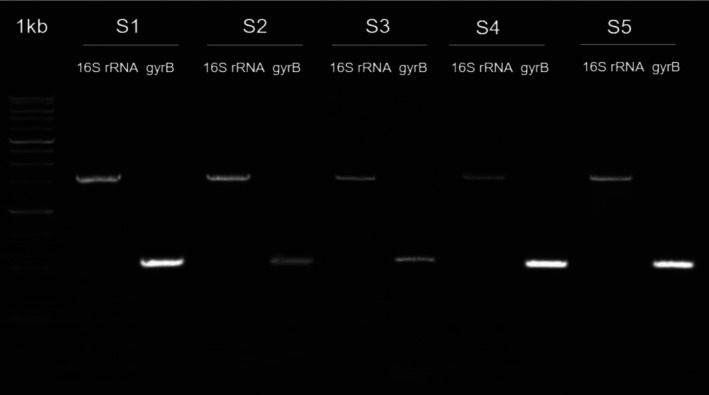
DNA band image of PCR electrophoresis results from 5 samples. Description: S1 = Station 1, S2 = Station 2, S3 = Station 3, S4 = Station 4, S5 = Station 5.

Figure 3 shows the results of agarose gel electrophoresis from PCR amplification of two genes, 16S rRNA and gyrB, in five different samples (S1–S5). The upper bands correspond to the 16S rRNA gene, while the lower bands represent the gyrB gene. All samples showed strong and clear bands for 16S rRNA, indicating successful amplification. Amplification of gyrB was also successful in S1, S2, S4 and S5, although the band in S2 appeared slightly fainter. In S3, the gyrB band was very weak or barely visible, suggesting inefficient amplification. The variation in gyrB band intensity may be due to differences in DNA quality, template concentration, or suboptimal PCR conditions. Overall, 16S rRNA amplification was successful in all samples, while gyrB requires further optimization, especially in sample S3.

After the DNA fragments are successfully amplified through PCR, the next step is sequencing, which involves reading the nucleotide sequence of the DNA. This technique aims to obtain detailed genetic information to identify virulence genes with pathogenic potential. The sequencing results are then analysed by aligning the DNA sequences with existing databases from the National Center for Biotechnology Information (NCBI). Using BLAST (Basic Local Alignment Search Tool), the query sequences are compared to database entries to identify sequence similarities and determine the specific organism or gene, and the results are in Table 4.

Based on the molecular identification results (Table 4) using the gyrB and 16S rRNA genes, two main *Vibrio* species were detected from the five isolates analysed, namely *V. alginolyticus* (S2, S3, S4) and *V. parahaemolyticus* (S1, S5). The sequence similarity values of the gyrB gene were higher (96.71%–99.05%) compared to those of the 16S rRNA gene (94.55%–98.94%), indicating that gyrB is more sensitive and accurate in distinguishing species within the *Vibrio* genus. The consistency of identification between the two genes was observed in S1 and S5, which showed a close relationship with *V. parahaemolyticus* , as well as in S2–S4, which were closely related to *V. alginolyticus* . Nevertheless, the relatively lower similarity percentages in the 16S rRNA gene may pose limitations in species resolution.

**TABLE 4:** BLAST analysis of 16S rRNA and gyrB sequences of Vibrio Species Isolated from Vannamei Shrimp.

Sample codes	Closest species relative	Gene			
		gyrB		16S rRNA	
		Similarity	Genbank accession	Similarity	Genbank accession
S1	*Vibrio parahaemolyticus*	96.71%	CP078613.1	98.94%	CP078613.1
S2	*Vibrio alginolyticus*	98.58%	CP110670.1	95.22%	CP110670.1
S3	*Vibrio alginolyticus*	97.95%	CP017899.1	95.86%	CP017899.1
S4	*Vibrio alginolyticus*	96.99%	CP017911.1	96.39%	CP017911.1
S5	*Vibrio parahaemolyticus*	99.05%	CP150909.1	94.55%	CP013484.1

**FIGURE 4:**
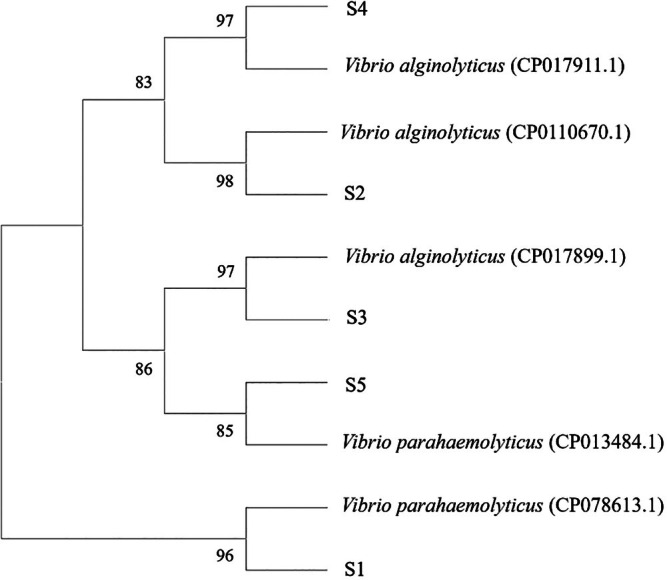
Phylogenetic tree of *gyrB* Genes of Vibrio species isolated from Vannamei Shrimp (1000× bootstrapping).

Based on sequencing results, the selected samples were analysed using BioEdit and MEGA 11 software to construct a phylogenetic tree, as shown in Figure 4. This phylogenetic tree was generated using relevant gene sequences, with bootstrap values at each branch indicating the confidence level for the branching points. Higher bootstrap values provide stronger statistical support for the relatedness among species. In general, isolates S2, S3 and S4 exhibited a very close genetic relationship with several strains of *V. alginolyticus* , as indicated by high bootstrap values (> 96). Specifically, isolate S2 clustered with *V. alginolyticus* strain CP110670.1 with a bootstrap value of 98, isolate S3 formed a clade with *V. alginolyticus* strain CP017899.1 supported by a bootstrap value of 97, and isolate S4 grouped with *V. alginolyticus* strain CP017911.1 with a bootstrap value of 97.

In contrast, isolates S1 and S5 were positioned on a separate branch and showed a close relationship with *V. parahaemolyticus* . Isolate S1 clustered with *V. parahaemolyticus* strain CP078613.1 with a bootstrap value of 96, while isolate S5 formed a clade with *V. parahaemolyticus* strain CP013484.1 supported by a bootstrap value of 85. These results suggest that isolates S1 and S5 belong to *V. parahaemolyticus* , whereas isolates S2, S3 and S4 are members of *V. alginolyticus* .

### 3.1 Determination of Toxin Gene

**FIGURE 5:**
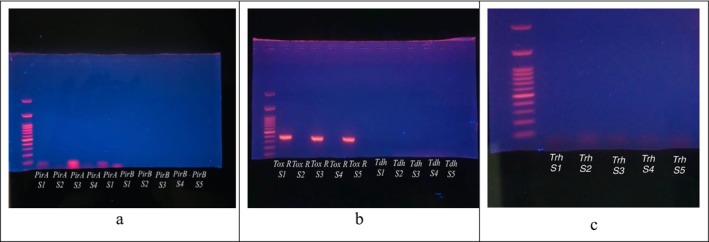
Electrophoresis determination of toxin gene. (a) PCR product of gene PirA and PirB. (b) PCR product of gene Tox R and Tdh. (c) PCR product of gene Trh.

The detection of toxin genes in *Vibrio* isolates from shrimp samples (Table 5, Figure 5) revealed that among the five genes tested (*tdh*, *trh, toxR*, *pirA* and *pirB*), only the toxR gene was found to be positive. Clear amplification bands of the *toxR* gene were observed in isolates S1 ( *V. parahaemolyticus* ), S3 ( *V. alginolyticus* ) and S5 ( *V. parahaemolyticus* ), whereas isolates S2 and S4 ( *V. alginolyticus* ) were negative. No amplification was detected for *tdh*, *trh*, *pirA* and *pirB* across all isolates. These results indicate that the strains identified in this study are not clinical types associated with human gastroenteritis or AHPND; however, the presence of the *toxR* gene remains significant as it serves as a major regulator of virulence factors in *Vibrio* spp. This finding is also consistent with the clinical symptoms observed in the field, particularly at Station 1, which were closely associated with red disease in shrimp.

**TABLE 5:** Detection of toxin genes in Vibrio isolates from shrimp samples.

No	Sample codes	Closest species relative	Genes				
			toxR	tdh	trh	pirA	pirB
1	S1	*Vibrio parahaemolyticus*	+	−	−	−	−
2	S2	*Vibrio alginolyticus*	−	−	−	−	−
3	S3	*Vibrio alginolyticus*	+	−	−	−	−
4	S4	*Vibrio alginolyticus*	−	−	−	−	−
5	S5	*Vibrio parahaemolyticus*	+	−	−	−	−

### 3.2 Quantitative Assessment of TVC (Total Vibrio Count) in Shrimp

**FIGURE 6:**
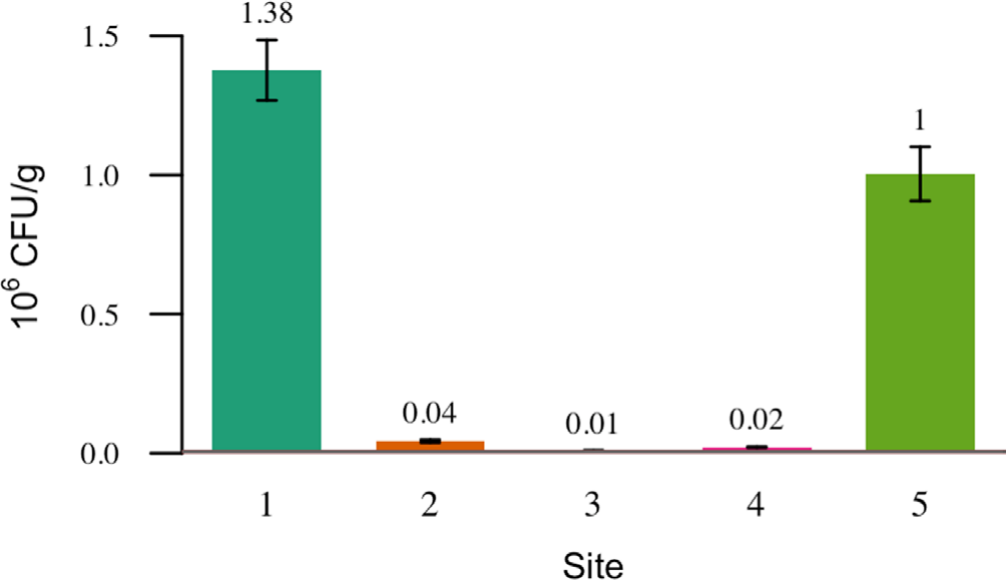
TVC results of bacteria on Pangandaran coast on Shrimp.

The analysis results showed that the highest TVC value was found at Site 1, which was 1.3767 × (10^6^) CFU/g, followed by Site 5 with a value of 1.0043 × 10^6^ CFU/g (Figure 6). ANOVA results indicated a statistically significant difference in bacterial abundance among the sites (*p* < 0.05), confirming that the variations observed were not due to random chance. Post hoc Tukey HSD analysis further revealed that Site 1 and Site 5 had significantly higher levels of *Vibrio* bacteria compared to other locations, suggesting a greater potential risk of disease if proper management is not implemented. Conversely, the lowest TVC was recorded at Site 3, at 0.009 × 10^6^ CFU/g, indicating that environmental conditions or aquaculture practices such as the use of probiotics may have been more effective in suppressing *Vibrio* populations.

### 3.3 Proportional Assessment of Vibrio and Non‐Vibrio Bacteria in Shrimp

**FIGURE 7:**
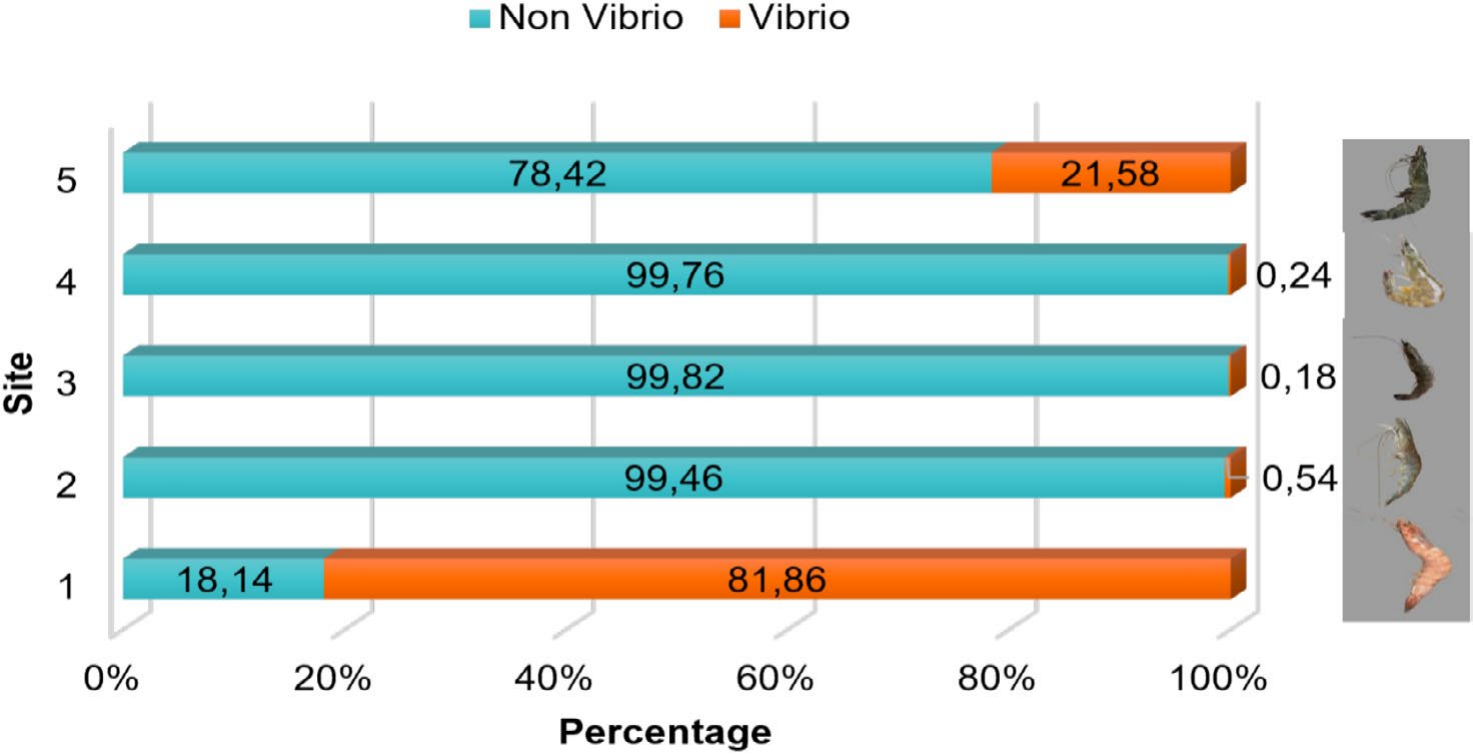
Proportion of the number of Vibrio and Non‐Vibrio bacteria in Shrimp.

Figure 7 shows the percentage distribution of bacterial growth categorized into *Vibrio* and *non‐Vibrio* groups across five study locations. At Station 1, there was a dominance of *Vibrio* bacteria, with a percentage of 81.86%, indicating contamination by pathogenic *Vibrio* species that could cause diseases in shrimp. Only 18.14% of bacteria were classified as *non‐Vibrio*. At Stations 2, 3 and 4, the bacterial composition was dominated by *non‐Vibrio* species, ranging from 99.46% to 99.82%, while *Vibrio* bacteria were nearly absent, ranging only from 0.18% to 0.54%.

### 3.4 Shrimp Diseases

According to the Fisheries and Marine Affairs Department of Pangandaran Regency, five bacterial diseases caused by *Vibrio* spp. (vibriosis) have been reported in shrimp ponds in Pangandaran, West Java. These included Acute Hepatopancreatic Necrosis Disease (AHPND), Infectious Myonecrosis Virus (IMNV), White Spot Syndrome Virus (WSSV), White Faeces Disease (WFD), Septic Hepatopancreatic Necrosis Disease (SHND), and Red Disease.

**FIGURE 8:**
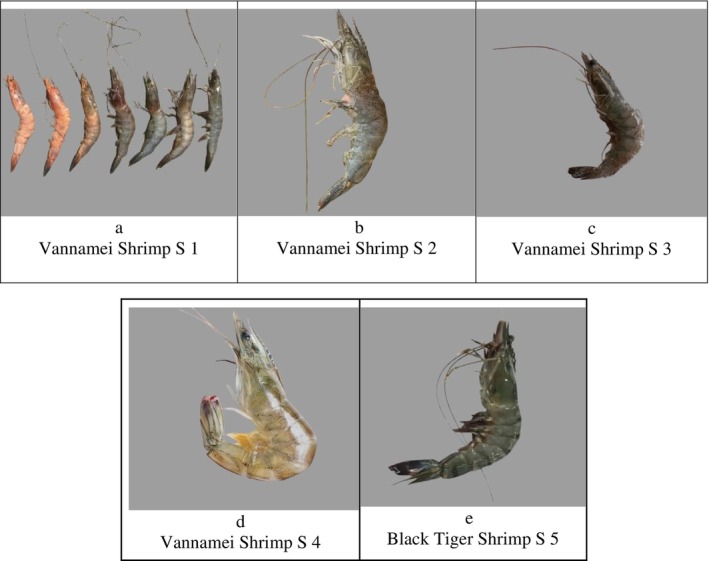
(a) Farmed whiteleg shrimp ( *Litopenaeus vannamei* ) infected by *Vibrio* sp. showing reddish body colour change. (b–d) Normal whiteleg shrimp ( *Litopenaeus vannamei* ) and (e) Normal tiger shrimp ( *Penaeus monodon* ).

Shrimp in Figure 8a showed abnormal physical characteristics, such as a red discolouration, which indicated bacterial infection or environmental stress. According to the pond owner, shrimp also showed sluggish movement, which was a clear sign of health problems (Table 6). At Stations 2, 3, 4 and 5, shrimp were found to be in normal condition without any concerning clinical signs. This showed that there were no health issues found in shrimp at the stations.

**TABLE 6:** Clinical symptoms, behavioural changes, and gross lesions.

Station	Clinical symptoms	Behavioural changes	Rough lesions
1	Reddish body colour	Slower moving	Red spots on body parts
2	Normal	Normal	Normal
3	Normal	Normal	Normal
4	Normal	Normal	Normal
5	Normal	Normal	Normal

**FIGURE 9:**
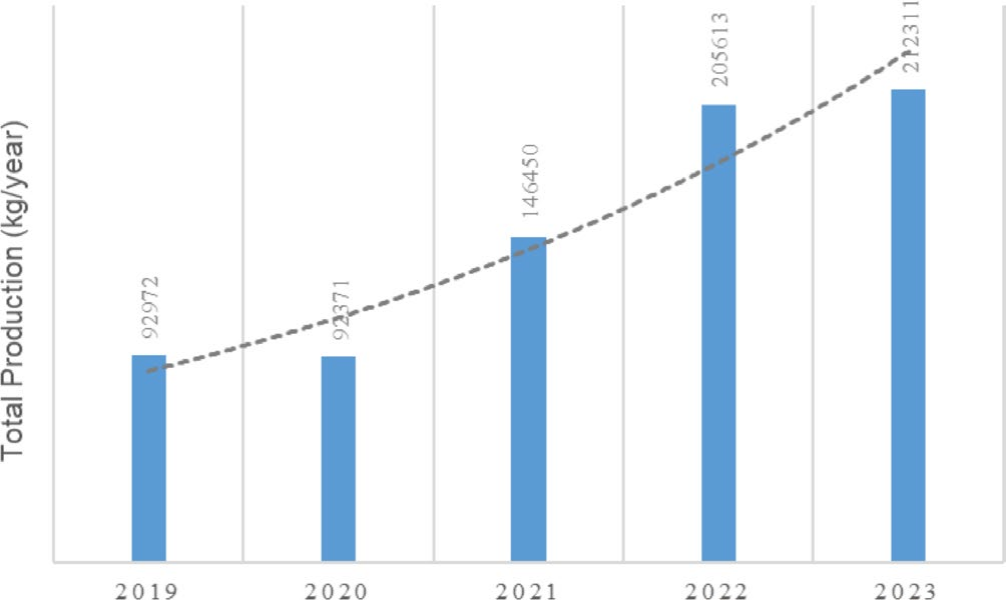
Total production of Shrimp from farming in Ponds in Pangandaran Regency. *Source:* Department of Marine and Fisheries of Pangandaran Regency 2024.

According to the report from the Department of Marine and Fisheries of Pangandaran Regency, shrimp production in 2020 slightly decreased from 92,972 kg/year to 92,371 kg/year (Figure 9), influenced by environmental factors, weather conditions, and farming practices. However, production significantly increased in 2021 to 146,450 kg/year and continued to rise in 2022 and 2023, indicating improvements in farming methods and pond management.

## 4 Discussion

Thiosulfate citrate bile salt sucrose (TCBS) agar was used due to its selective properties for *Vibrio*, producing yellow or green colonies with distinctive morphology (Oliver and Kaper 2001). Species identification was conducted using PCR targeting the 16S rRNA and gyrB genes, which are conserved among *Vibrio* species (Watanabe et al. 2001), and further confirmed by sequencing to ensure rapid and accurate results. In addition, toxin gene detection was performed to evaluate the virulence potential of the isolates, including tdh (thermostable direct hemolysin), trh (TDH‐related hemolysin), toxR (toxin operon regulator), as well as pirA and pirB, which are associated with Acute Hepatopancreatic Necrosis Disease (AHPND).

*Vibrio* are Gram‐negative, rod‐ or comma‐shaped bacteria that proliferate in high‐salinity environments (Madigan et al. 2004). Of more than 140 known species, 12 are pathogenic to humans and 16 are capable of infecting aquatic animals (Mohamad et al. 2019; Cuéllar‐Ánjel et al. 2014; Beaz‐Hidalgo et al. 2010). Infections typically occur when hosts experience stress due to environmental fluctuations (Defoirdt et al. 2004), although certain species, such as those causing AHPND, can infect even in the absence of stress conditions (Drake et al. 2007). Overfeeding has also been reported to exacerbate infections.

Previous environmental monitoring in Pangandaran (Herawati, Faddilah, et al. 2025) focused on the identification and quantification of bacterial communities in pond water, seawater and sediment across the same five stations examined in this study. That research showed that bacterial loads, particularly on TCBS medium, were highest in pond sediments, with *V. alginolyticus* and *V. fluvialis* detected in intensive and semi‐intensive pond sediments. Conversely, pond water and seawater samples were dominated by *Bacillus* spp., including *Bacillus flexus* and *Bacillus albus* , which are often associated with probiotic activity and improved pond health. The bacterial composition of sediments indicated a higher risk of serving as reservoirs for pathogenic *Vibrio*, due to the accumulation of organic matter from feed residues and faeces, thereby creating eutrophic micro‐environments that promote *Vibrio* proliferation.

By integrating these environmental data with host tissue‐based findings from the present study, a clearer picture emerges: sediments reported previously function as reservoirs of pathogenic *Vibrio*, whereas direct isolation from shrimp tissues in this study revealed strains that actively colonise the host. For example, *V. alginolyticus* detected in sediments at Station 4 was also isolated from shrimp tissues in this study, albeit without clear clinical symptoms suggesting possible subclinical infection or low virulence under effective farm management practices (e.g., probiotic application by farmers). In contrast, at Station 1, the presence of *V. parahaemolyticus* in shrimp tissues coincided with the high nutrient loads previously reported in surrounding waters and sediments, alongside the occurrence of disease symptoms in this study. This highlights a potential link between environmental reservoirs of *Vibrio*, water quality and active infections in shrimp.

The identification results based on colony morphology and PCR indicated the presence of *V. parahaemolyticus* at Station 1 (Table 4). Early harvesting was carried out due to the emergence of disease symptoms in shrimp (Figure 8a). The clinical signs observed in this study showed a strong association with red disease, an important condition in shrimp aquaculture caused by bacterial infections, particularly from the genera *Vibrio* and *Providencia*. This disease is mainly attributed to pathogenic *Vibrio* species such as *V. harveyi* , *V. parahaemolyticus* , *V. alginolyticus* and *V. campbellii* (Guzman et al. 2022; Haifa‐Haryani et al. 2022; Yasin et al. 2018). Red disease has been reported to infect major cultured shrimp species, including *Marsupenaeus japonicus* and *Penaeus vannamei* (Cao et al. 2018, 2022; Haifa‐Haryani et al. 2022).

The clinical signs of red disease observed in this study were consistent with previous reports. The shrimp exhibited reddish discolouration on the uropods, telson and abdominal segments (Figure 8a), accompanied by reddish‐brown gills and changes in the hepatopancreas. In severe cases, the hepatopancreas appeared pale, indicating an advanced stage of infection (Sarjito et al. 2022; Setyastuti et al. 2023). In addition, the infected shrimp appeared lethargic, lost appetite and showed reduced swimming activity (Table 6).

The progression of the disease occurred gradually, beginning with mild symptoms that later developed into severe conditions and could eventually cause mass mortality if not promptly managed. The stages of red disease symptoms observed in this study were consistent with the description of Alapide‐Tendencia and Dureza (1997), which include changes in body colouration from yellowish‐green, to reddish and finally to bright red (Figure 8a). At the terminal stage, shrimp appeared weak, ceased feeding and their body surfaces became colonised by epibiotic organisms.

Environmental factors such as poor water quality, hypoxia, high stocking density and elevated water temperature greatly contributed to the onset and severity of this disease (Ghani et al. 2024; Stentiford et al. 2012).

The PCR electrophoresis results showed that among the five toxin genes tested (*tdh, trh*, *toxR*, *pirA* and *pirB*), only the toxR gene was detected as positive. The absence of *tdh* and *trh* indicates that the tested *V. parahaemolyticus* strain is not a clinical type associated with gastroenteritis in humans. Similarly, the lack of *pirA* and *pirB* differentiates this case from Acute Hepatopancreatic Necrosis Disease (AHPND), which is classically caused by *V. parahaemolyticus* strains producing PirA and pirB toxins (Han et al. 2015; Restrepo et al. 2016; Sirikharin et al. 2015).

Nevertheless, the detection of toxR remains significant, as this gene is a major regulator that controls the expression of various virulence factors in *V. parahaemolyticus* , including TDH and TRH hemolysins, and plays a crucial role in pathogenic mechanisms affecting aquatic hosts (Han et al. 2015; Yang et al. 2017). Previous studies have confirmed that *V. parahaemolyticus* strains carrying the toxR gene exhibit a higher level of pathogenicity compared to strains lacking this gene (Luangtrakul et al. 2021; Yang et al. 2017).

Furthermore, several studies have reported a direct association between the presence of the toxR gene and the manifestation of red disease in shrimp. The clinical symptoms of red disease, such as reddish discolouration of the uropods, telson and gills, are consistent with field observations in this study. Asgarpoor et al. (2018) showed that *Vibrio* isolates carrying the toxR gene were dominant in shrimp samples exhibiting disease symptoms, thereby confirming the strong link between this gene and pathogenicity. This is further supported by Wang et al. (2020), who found that hemolytic activity in *Vibrio* strains is associated with the presence of the toxR gene, which contributes to gill tissue damage in shrimp. In addition to *V. parahaemolyticus* , other species such as *V. alginolyticus* have also been reported to harbour virulence genes, including toxR, and are potentially capable of triggering similar symptoms.

The aquaculture environment further exacerbates disease manifestation. Factors such as poor water quality, hypoxia and high stocking density can increase shrimp susceptibility to *Vibrio* infections (Alfiansah et al. 2018; Du et al. 2024; Satyantini et al. 2024). The complex interaction between environmental factors and bacterial communities has also been described by Cao et al. (2022), who reported that environmental stress can accelerate the conversion of *Vibrio* strains from non‐virulent to virulent forms.

Thus, although the strains identified in this study did not carry the pirA and pirB genes responsible for AHPND, the presence of the toxR gene still indicates a virulence potential relevant to the pathogenesis of red disease. These findings highlight the need for improved aquaculture management, including stricter water quality control and the implementation of targeted immunological strategies to mitigate disease risk (Dhar et al. 2019; Sugiharta et al. 2023).

Analysis results showed that the highest Total *Vibrio* Count (TVC) was found at Station 1, amounting to 1.3767 × 10^6^ CFU/g (Figure 6). Moreover, the proportion of *Vibrio* bacteria was more dominant than non‐*Vibrio* bacteria (Figure 7), indicating a high presence of pathogenic halophilic bacteria at this site.

This condition is related to the poor water quality at Station 1 in the same study location, which did not meet the standard limits. According to Herawati, Rahayu, et al. (2025), total ammonia concentration was recorded at 0.52 ± 0.02 mg/L, nitrate at 0.78 ± 0.43 mg/L and phosphate at 0.27 ± 0.02 mg/L, all exceeding the quality standards set by Government Regulation of the Republic of Indonesia No. 22 of 2021. Such an environment rich in inorganic nutrients supports the proliferation of *Vibrio* spp., especially under eutrophic conditions and moderate salinity (Arisandi et al. 2017). The high total bacteria and dominance of *Vibrio* at Station 1 reflect a high potential for disease outbreaks. This led to the appearance of disease symptoms in shrimp and prompted early harvesting as a preventive measure. Therefore, mitigation efforts are needed through water quality management and the implementation of sustainable aquaculture practices.

Observations at Stations 2, 3 and 4 showed the presence of *V. alginolyticus* in shrimp samples. Morphological identification showed distinct colony colours on TCBS media, and PCR results targeting the *gyrB* gene confirmed the presence of the bacteria (Table 4). Although *V. alginolyticus* is part of the normal marine flora (Slifka et al. 2017), it is also known as a major pathogen causing vibriosis, which can lead to skin lesions, swelling and sudden death in shrimp.

At these three stations, despite the detection of *V. alginolyticus* , the abundance of *Vibrio* bacteria was very low (Figure 6), and their proportion was also smaller compared to non‐*Vibrio* bacteria (Figure 7). The tested shrimp appeared healthy and showed no clinical symptoms (Figure 8b–d). According to Herawati, Faddilah, et al. (2025), water quality in the three locations showed nutrient concentrations, particularly nitrate and phosphate, exceeding the thresholds. At Station 2, nitrate was recorded at 0.92 ± 0.45 mg/L and phosphate at 0.04 ± 0.02 mg/L; Station 3 had nitrate at 0.44 ± 0.18 mg/L and phosphate at 0.07 ± 0.02 mg/L; while Station 4 showed nitrate at 0.55 ± 0.42 mg/L and phosphate at 0.10 ± 0.10 mg/L. Nonetheless, other parameters such as BOD were still within safe limits.

Overall, despite the increased nutrient concentrations, they did not significantly impact shrimp health. The use of probiotics and good water quality management is believed to play important roles in maintaining microbial balance and preventing clinical symptoms. Regular monitoring is still required to support the sustainability of the culture system.

Shrimp samples from the Karapyak waters at Station 5, specifically tiger shrimp caught by local fishers, showed a *Vibrio* percentage of 21.58%. This indicates the presence of *Vibrio* in the marine environment, while the remaining 78.42% consisted of non‐*Vibrio* bacteria (Figure 6). The bacteria identified in the wild‐caught tiger shrimp included *V. parahaemolyticus* (Table 4). These bacterial species are known pathogens that can cause various diseases in shrimp.

In these tiger shrimp samples, no signs of infection or other clinical symptoms were found (Figure 8e), although the *Vibrio* count was relatively high (Figure 6). Sha et al. (2016) stated that wild‐caught tiger shrimp show better immune responses to infections compared to cultured shrimp. This stronger immune response is linked to the natural environment, where shrimp exposed to natural microorganisms in their habitat tend to develop more effective immune systems against pathogens such as *Vibrio*. Exposure to diverse microorganisms in a biodiverse marine ecosystem may influence the shrimp's immune system and enhance its resistance to pathogen attacks. These results highlight the importance of natural environmental conditions in supporting shrimp health and resistance to disease, particularly in maintaining the sustainability of aquatic ecosystems.

This study demonstrates that *Vibrio* contamination in shrimp from coastal ponds in Pangandaran poses varying disease risks. At Station 1, *V. parahaemolyticus* was dominant and linked to clinical signs of red disease, while *V. alginolyticus* at Stations 2–4 appeared in low abundance without symptoms. In wild tiger shrimp from Station 5, *V. parahaemolyticus* was detected but not associated with disease. The presence of the *toxR* gene in several isolates indicates potential virulence, although the absence of *tdh*, *trh*, *pirA* and *pirB* suggests the strains were not highly pathogenic or AHPND‐causing types.

Despite increasing production (Figure 9), the persistence of pathogenic *Vibrio* remains a major challenge for sustainability. Effective control requires integrated health management, including strict biosecurity, safe disposal of infected shrimp, continuous disease surveillance and prevention of cross‐contamination. Maintaining water quality, lowering stocking density, reducing environmental stress and ensuring adequate nutrition are also essential to minimize infection risks and support long‐term pond productivity (Lavilla‐Pitogo and de la Peña 2004; Cuéllar‐Ánjel et al. 2014; Radhakrishnan and Kizhakudan 2019).

## 5 Conclusion

This study demonstrated the presence of *Vibrio* and non‐*Vibrio* bacteria in *Litopenaeus vannamei* and *Penaeus monodon* at five coastal stations in Pangandaran. The identified *Vibrio* species, *V. parahaemolyticus* and *V. alginolyticus* , were suspected to be associated with red disease, with the highest dominance observed at Station 1 (Kertamukti), where vannamei shrimp showed clinical symptoms that led to early harvesting. In contrast, wild tiger shrimp at Station 5 (Karapyak) remained healthy despite harbouring *Vibrio* spp. Toxin gene analysis revealed that only the toxR gene was detected, while *tdh, trh, pirA* and *pirB* were absent. This indicates that the isolates were not human‐pathogenic clinical types or AHPND‐causing strains, but the presence of *toxR* suggests potential virulence associated with shrimp infections such as red disease. These findings provide an important basis for environmental assessment and the development of mitigation policies to reduce *Vibrio* contamination in shrimp aquaculture in Pangandaran. Strengthening collaboration in the implementation of mitigation strategies is required to support the sustainability of shrimp production.

## Author Contributions

**Titin Herawati:** conceptualization, writing – original draft preparation, writing – review and editing, methodology, funding, investigation, resources, supervision. **Indriyani Rahayu:** writing – original draft preparation, writing – review and editing, data curation, formal analysis, resources, methodology, software, validation. **Aisyah Aisyah:** writing – original draft preparation, writing – review and editing, data curation, formal analysis, methodology, software, validation, resource. **Mochamad Untung Kurnia Agung:** methodology, validation, reviewing and editing, resources. **Buntora Pasaribu:** writing – reviewing and editing, resources. **Atikah Nurhayati:** reviewing, resources. **Adiana Ghazali:** reviewing and editing, resources. **Roffi Grandiosa:** reviewing, resources. **Thallita Nasywa Faddilah:** resources, project administration. **Rendika Kamiswara:** validation, resources.

## Conflicts of Interest

The authors declare no conflicts of interest.

## Data Availability

The data that support the findings of this study are available on request from the corresponding author. The data are not publicly available due to privacy or ethical restrictions.
